# Bladder cancer – the neglected tumor: a descriptive analysis of publications referenced in MEDLINE and data from the register clinicaltrials.gov

**DOI:** 10.1186/1471-2490-13-56

**Published:** 2013-10-24

**Authors:** Frank Kunath, Steffen F Krause, Bernd Wullich, Peter J Goebell, Dirk G Engehausen, Maximilian Burger, Joerg J Meerpohl, Bastian Keck

**Affiliations:** 1 Department of Urology, University Hospital Erlangen, Krankenhausstr. 12, 91054 Erlangen Germany; 2 Department of Urology, General Hospital Linz, 4021 Linz Austria; 3 Department of Urology and Pediatric Urology, Julius-Maximilians-University Medical Center, 97080 Würzburg, Germany; 4 German Cochrane Centre, Department of Medical Biometry and Medical Informatics, University Medical Centre Freiburg, Freiburg, Germany

**Keywords:** Kidney neoplasms, Prostatic neoplasms, Randomized controlled trial, Testicular neoplasms, Urinary bladder neoplasms

## Abstract

**Background:**

Uro-oncological neoplasms have both a high incidence and mortality rate and are therefore a major public health problem. The aim of this study was to evaluate research activity in uro-oncology over the last decade.

**Methods:**

We searched MEDLINE and ClinicalTrials.gov systematically for studies on prostatic, urinary bladder, kidney, and testicular neoplasms. The increase in newly published reports per year was analyzed using linear regression. The results are presented with 95% confidence intervals, and a *p* value <0.05 was considered statistically significant.

**Results:**

The number of new publications per year increased significantly for prostatic, kidney and urinary bladder neoplasms (all <0.0001). We identified 1,885 randomized controlled trials (RCTs); also for RCTs, the number of newly published reports increased significantly for prostatic (p = 0.001) and kidney cancer (p = 0.005), but not for bladder (p = 0.09) or testicular (p = 0.44) neoplasms. We identified 3,114 registered uro-oncological studies in ClinicalTrials.gov. However, 85% of these studies are focusing on prostatic (45%) and kidney neoplasms (40%), whereas only 11% were registered for bladder cancers.

**Conclusions:**

While the number of publications on uro-oncologic research rises yearly for prostatic and kidney neoplasms, urothelial carcinomas of the bladder seem to be neglected despite their important clinical role. Clinical research on neoplasms of the urothelial bladder must be explicitly addressed and supported.

## Background

Uro-oncological neoplasms have both a high incidence and mortality rate [1,2]. Urological neoplasms are therefore a major public health problem [3]. The treatment of prostatic neoplasms costs 11.85 billion dollars, representing 15% of the average annual cost for the treatment of all cancers in the United States (http://www.cancer.gov). In 2010, the treatment cost for urinary bladder and renal cancers in the United States was 3.98 billion and 3.80 billion dollars, respectively (http://www.cancer.gov). Therefore, uro-oncological research should pursue two major goals. On the one hand, patient-centered care must be optimized; on the other hand, it must be regulated effectively. Generating high quality studies is indispensable to provide treatments based on the best clinical evidence available. However, it is not clear whether current scientific research output adequately addresses clinical requirements of this population. The aim of this study was to investigate current research activity in the field of uro-oncology.

## Methods

This analysis focused on scientific publications in the field of uro-oncology. Where appropriate, the systematic literature search was designed, conducted, and reported in accordance with the Preferred Reporting Items for Systematic Reviews and Meta-Analysis (PRISMA) [4].

First, we searched MEDLINE (Ovid MEDLINE® 1946 to Present with daily update; searched 31.08.2012), an electronic database of biomedical literature, for any reports on the four relevant urological tumors with high incidences and mortality rates (prostatic, urinary bladder, kidney, and testicular neoplasms) based on recent data publically available from the International Agency for Research on Cancer of the World Health Organization [1]. We used medical subject heading (MeSH) terms on prostatic (prostatic neoplasms/), kidney (kidney neoplasms/), urinary bladder (urinary bladder neoplasms/) and testicular neoplasms (testicular neoplasms/). The search included all reports published from 2001 to 2011. Then, we narrowed our search strategy by limiting it to randomized controlled trials, clinical controlled trials, meta-analyses, and case reports using a limit function provided by MEDLINE ('publication type’). For definitions of the urological tumors provided by MEDLINE see Table 1. The search strategy for reports on 'urinary bladder neoplasm/’ in MEDLINE is displayed as an example in Table 2.

**Table 1:** Definitions of the urological tumors as used by MEDLINE


Prostatic neoplasms	Tumors or cancers of the prostate
Kidney neoplasms	Tumors or cancers of the kidney
Urinary bladder neoplasms	Tumors or cancers of the urinary bladder
Testicular neoplams	Tumors or cancer of the testis, germ cell tumors (germinoma) of the testis constitute 95% of all testicular neoplasms

**Table 2:** Expanded search strategy for the MeSH term 'urinary bladder neoplasms’ in MEDLINE


Neoplasm, urinary bladder	Bladder neoplasm
Urinary bladder neoplasm	Neoplasm bladder
Bladder tumors	Urinary bladder cancer
Bladder tumor	Cancer, urinary bladder
Tumor, bladder	Cancer of bladder
Tumors, bladder	Cancer of the bladder
Neoplasms, bladder	Bladder cancer
Bladder neoplasms	Bladder cancers
Cancer, bladder	

Second, we searched the publically available trial register ClinicalTrials.gov for studies on the relevant urological neoplasms (http://www.clinicaltrials.gov; searched: 13.09.12). ClinicalTrials.gov is a registry for studies conducted in the United States and worldwide. It currently contains data sets on 132,156 trials originating from 179 countries (http://www.clinicaltrials.gov). We used the search function provided by ClinicalTrials.gov to identify studies on prostatic, kidney, urinary bladder and testicular neoplasms. The search function expands the search strategy according to an internal algorithm. The search strategy for studies on kidney cancer in ClinicalTrials.gov is displayed in Table 3 as an example. We searched for studies with start dates between 2005 and 2011 because prospective trial registration has been a requirement since July 1, 2005 only (http://www.icmje.org).

**Table 3:** Expanded search strategy for urinary bladder neoplasms in ClinicalTrials.gov


Bladder ca	Tumor of urinary bladder
Bladder cancer	Urinary bladder malignant tumor
Bladder carcinoma	Tumor of the urinary bladder
Bladder neoplasms	Tumor of the bladder
Bladder tumors	Tumor of bladder
Cancer of bladder	Tumor bladder
Cancer of the bladder	Neoplasm of urinary bladder
Cancer of the urinary bladder	Neoplasm of the urinary bladder
Cancer of urinary bladder	Neoplasm of the bladder
Carcinoma of bladder	Neoplasm of bladder
Carcinoma of the bladder	Malignant neoplasm of the bladder
Carcinoma of the urinary bladder	Carcinoma of urinary bladder

We evaluated the increase of newly published reports per year using linear regression and presented all results with 95% confidence intervals (CI). Statistical analysis was performed with the IBM SPSS Statistics 19 software package. All of the tests were two-sided, and a *p* value <0.05 was considered statistically significant (SPSS 19 software, IBM SPSS, Chicago, IL).

## Results

**Figure 1:**
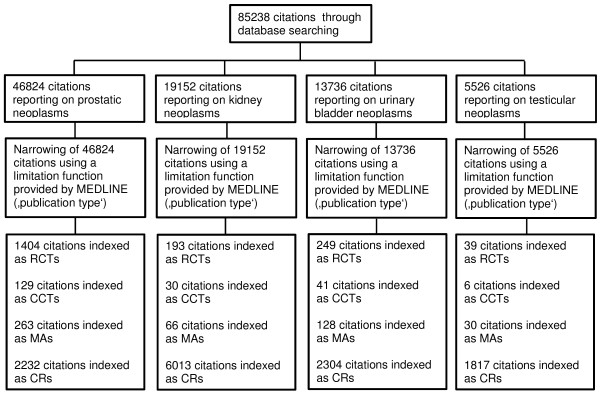
**Search flow chart (Ovid MEDLINE®).** RCTs, randomized controlled trials; CCTs, clinical controlled trials; MAs, meta-analyses; CRs, case reports.

Our search strategy identified 85,238 uro-oncological citations in MEDLINE published between 2001 and 2011 (for details see Figure 1). Of these, 46,824 (54.9%) reported on prostatic neoplasms, 19,152 (22.5%) reported on kidney neoplasms, 13,736 (16.1%) reported on bladder neoplasms, and 5,526 (6.5%) reported on testicular neoplasms. The number of new publications per year increased significantly over the evaluated time period for prostatic, kidney and urinary bladder neoplasms (all <0.0001). Citations on prostatic neoplasms increased the most, with a median increase of 250.7 more publications than the year before (95% CI 214.2-287.2). There were 2828 citations on prostatic neoplasms in 2001 and 5666 in 2011. Interestingly, new reports on kidney cancer increased with a median increase of 106 more new publications per year (95% CI 96.3-115.6) far more than the increase in reports on urinary bladder cancer (40.8 more new publications/year, 95% CI 33.5-48.1). We identified no significant increase in the number of publication of reports on testicular neoplasms (median 6 more new publications/year, 95% CI -3.2-15.2, p = 0.17; Table 4).

**Table 4:** Uro-oncological publications in Ovid MEDLINE (2001–2011)

	**Total**	**Increase of new publications per year**	**p value**
		**(95% Confidence interval)**	
Total publications			
Prostatic neoplasms	46824	250.7 (214.2-287.2)	<0.0001
Kidney neoplasms	19152	106 (96.3-115.6)	<0.0001
Urinary bladder neoplasms	13736	40.8 (33.5-48.1)	<0.0001
Testicular neoplasms	5526	6 (-3.2-15.2)	0.17
Publications indexed as randomized controlled trials			
Prostatic neoplasms	1404	8.1 (4.2-12)	0.001
Kidney neoplasms	193	1.9 (0.7-3)	0.005
Urinary bladder neoplasms	249	0.5 (-0.2-2.4)	0.09
Testicular neoplasms	39	0.1 (-0.2-0.5)	0.44
Publications indexed as clinical controlled trials			
Prostatic neoplasms	129	0.2 (-1.6-1.2)	0.76
Kidney neoplasms	30	-0.3 (-0.6-0.1)	0.12
Urinary bladder neoplasms	41	-0.4 (-0.8-0)	0.05
Testicular neoplasms	6	-0.1 (-0.2-0)	0.15
Publications indexed as meta-analysis			
Prostatic neoplasms	263	3 (2–3.9)	<0.0001
Kidney neoplasms	66	1.3 (0.7-1.9)	0.001
Urinary bladder neoplasms	128	1.6 (0.6-2.6)	0.006
Testicular neoplasms	30	0.4 (-0.1-0.8)	0.06
Publications indexed as case reports			
Prostatic neoplasms	2232	0.5 (-3.5-4.4)	0.8
Kidney neoplasms	6013	22.6 (15.6-29.5)	<0.0001
Urinary bladder neoplasms	2304	0 (-3.4-3.5)	0.98
Testicular neoplasms	1817	0 (-4.3-4.4)	0.99

The limitation focusing on published reports of studies indexed in MEDLINE as randomized controlled trials revealed 1,885 studies published between 2001 and 2011: 1404 (74.5%) on prostatic neoplasms, 193 (10.2%) on kidney neoplasms, 249 (13.2%) on urinary bladder neoplasms, and 39 (2.1%) on testicular neoplasms. For details see Figure 1. While the number of newly published citations per year on randomized controlled trials increased significantly for reports on prostatic (p = 0.001) and kidney cancer (p = 0.005), we identified no significant differences among publications on urinary bladder (p = 0.09) and testicular (p = 0.44) neoplasms. With an approximate increase of 8 more new citations per year, the majority of randomized controlled trials were published on prostatic neoplasms (Table 4).

The number of publications of uro-oncological studies indexed in MEDLINE as clinically controlled trials was marginal and showed no statistical significant increase of new publications per year for any tumor entity. Remarkably, the number of publications on urinary bladder cancer showed a decreasing trend per year, but the decrease was not statistically significant (p = 0.05). The numbers of publications per year of reports indexed as meta-analyses increased significantly for prostatic (p < 0.0001), kidney (p = 0.001), and urinary bladder neoplasms (p = 0.006). However, the discovered maximum median increase of 3 more new meta-analyses per year for prostatic neoplasms is quite small. Interestingly, the numbers of new case-reports on kidney neoplasms amounted to a median increase of approximately 23 more new citations per year, a significant increase (p < 0.0001), while all other urological tumor entities remained on the same level over the investigated time period (Table 4).

**Figure 2:**
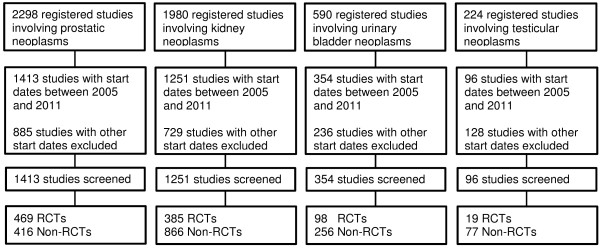
**Search flow chart (ClinicalTrials.gov).** RCTs, randomized controlled trials; Non-RCTs, non-randomized controlled trials.

We identified a total of 3,114 registered studies on prostatic, kidney, urinary bladder and testicular neoplasms with start dates between 2005 and 2011 from our search in ClinicalTrials.gov (Table 2). For details see Figure 2. Approximately 85% of these registered studies involved prostatic (1413 studies, 45%) or kidney neoplasms (1251 studies, 40%). Over a time period of 7 years (2005–2011), only 354 studies (11%) were registered on management of urinary bladder neoplasms. With 96 registered studies (3%), the quantity of entries on testicular cancers lagged far behind the other evaluated tumors. Approximately 30% of the total number of studies on prostatic (33.2%), kidney (30.8%) and urinary bladder neoplasms (27.7%) were registered as randomized controlled trials, while a much lower number (19.8%) of randomized controlled trials concerned testicular neoplasms (Table 5).

**Table 5:** Uro-oncological studies registered in ClinicalTrials.gov

	**Total**	**Randomized studies**	**Recruiting**	**Active, not recruiting**	**Not yet recruiting**	**Enrolling by invitation**	**Completed**	**Suspended**	**Terminated**	**Withdrawn**	Others*
Prostatic neoplasms	1413	469	536	300	23	20	399	11	99	23	2
		(33.2%)	(37.9%)	(21.2%)	(1.6%)	(1.4%)	(28.2%)	(0.8%)	(7%)	(1.6%)	(0.1%)
Kidney neoplasms	1251	385	518	247	26	15	317	12	86	27	3
		(30.8%)	(41.4%)	(19.7%)	(2.1%)	(1.2%)	(25.3%)	(1.0%)	(6.9%)	(2.2%)	(0.2%)
Urinary bladder neoplasms	354	98	159	63	5	6	89	0	23	9	0
		(27.7%)	(44.9%)	(17.8%)	(1.4%)	(1.7%)	(25.1%)	(0%)	(6.5%)	(2.5%)	(0%)
Testicular neoplasms	96	19	46	22	1	0	19	1	4	3	0
		(19.8%)	(47.9%)	(22.9%)	(1%)	(0%)	(19.8%)	(1%)	(4.2%)	(3.1%)	(0%)

* Available, approved for marketing, no longer available.

We also evaluated the status of uro-oncological studies as registered in ClinicalTrials.gov. However, we did not identify statistically significant differences between the various status classifications for each tumor entity (Table 5). We considered the statuses 'recruiting’, 'active, not recruiting’, 'not yet recruiting’, 'enrolling by invitation’, and 'completed’ as potentially positive for a subsequent analysis of the trial results; approximately 90% of the studies met these requirements (prostatic neoplasms: 90%; kidney neoplasms: 90%; urinary bladder neoplasms: 91%; testicular neoplasms: 92%). Only a minority of studies had 'suspended’, 'terminated’, or 'withdrawn’ statuses (prostatic neoplasms: 9.4%; kidney neoplasms: 10%; urinary bladder neoplasms: 9%; testicular neoplasms: 8%).

## Discussion

We analyzed the publication trends of the four relevant urological neoplasms and found that a tremendous increase of published uro-oncological literature has occurred over the past 10 years. However, the numbers of reports on urinary bladder neoplasms lag behind compared to kidney and prostate cancer research. Additionally, the vast majority of uro-oncological trials registered in ClinicTrials.gov were on prostatic and kidney neoplasms; only a small number addressed urinary bladder cancer.

We found that the total number of publications per year increased significantly for the combined urological tumor entities over the investigated time period. This development reflects the overall increase in the number of biomedical publications electronically available in databases such as MEDLINE. A total of 724,831 new citations were added to MEDLINE during 2011 [5]. With this rapidly increasing amount of knowledge, it is almost impossible for physicians to quickly filter out irrelevant evidence and to keep up-to-date with contemporary knowledge [6,7]. This difficulty is even exacerbated by the explosion of the types of publications currently available.

However, in contrast to these evaluations, we determined that the quantity of reports of randomized controlled trials for prostate and kidney cancer, which are indispensable for the evaluation of interventions, has increased significantly; however, for bladder and testicular neoplasms, this promising pattern was not clearly evident. This fact indicates that the prevalence of a tumor might not be reflected in the number of published reports of studies with a design not so prone to bias. Regarding the total number of publications in MEDLINE and registered studies in ClinicalTrials.gov, the level of research activity seems to be lower in bladder cancer compared to prostate cancer and renal cell carcinomas. Additionally, the publication of new uro-oncological studies indexed in MEDLINE as controlled clinical trials either was marginal or even declined in recent years. This development is in direct contrast to the clinical relevance of this tumor entity; it seems that the research activity on urothelial carcinoma has been overshadowed by scientific developments in kidney and prostate cancer research.

A recent evaluation by Bachir et al. also supports this conclusion [8]. The authors searched MEDLINE for randomized controlled trials published between 1995 and 2010. They concluded that only 238 randomized controlled trials had been published for bladder cancer over this time period. Furthermore, the quality of trials assessed was low. Only half of them had a sample size >100 patients, and only a small percentage of studies were double-blinded (8.0%). Additionally, less than one-third of the studies reported an appropriate power calculation [8]. The authors concluded that randomized controlled trials are therefore under-utilized in bladder cancer research [8]. This corresponds to our results, which highlight that urothelial bladder cancer is underrepresented in the literature compared to other urological tumor entities.

The number of uro-oncological citations indexed in MEDLINE as clinically controlled trials was marginal, and the number of meta-analyses on prostatic neoplasms per year is still very low. In certain clinical contexts, i.e., surgical therapy, it is not always feasible to take into account factors that might lower the potential risk of bias, such as blinding of participants and personnel. Thus, increasing the literature on prostatic neoplasms has become a crucial issue. Therefore, we agree with Bachir et al. in their demand for more randomized controlled trials with larger sample sizes to optimize the diagnostic and treatment strategies for patients with bladder cancer [8]. However, we believe this should not be limited to randomized controlled trials only. Also controlled clinical trials, systematic reviews and meta-analyses can contribute to the evidence-base that allows physicians optimally treat their patients.

We found no significant increase in the number of uro-oncological case reports on prostatic, urinary bladder or testicular cancer. The rising number of case reports on kidney cancer compared to other uro-oncologic tumors might be linked to the development of targeted therapies. The clinical implementation of these new drugs has provided completely new treatment options and allowed physicians to gain experience in detecting new adverse events. However, these results are usually published as case reports.

We also searched the publically available trial register ClinicalTrials.gov for uro-oncological studies with start dates between 2005 and 2011. Clinical Trials.gov is one of the most popular trial registers worldwide and contains more than 90,000 studies (http://www.clinicaltrials.gov). We found that approximately 85% of the identified uro-oncological studies were registered as involving prostatic or kidney neoplasms. As for urothelial neoplasms, the number of conducted and ongoing trials is far lower, which supports our observation that urothelial bladder neoplasms are neglected in the literature. The fact that 30% of the (few) identified studies on prostate, kidney and urothelial bladder neoplasms were randomized trials, however, is a potentially positive sign of the quality of uro-oncologic research; additionally, the frequency of randomized trials was just as high for urothelial bladder cancer. Only the number of randomized trials on testicular cancer showed a significantly lower number of reported trials (19.8%), which might reflect the low incidence of this tumor and its excellent clinical outcome due to current therapeutic approaches. Another positive finding is that 90% of the uro-oncological studies in ClinicalTrials.gov were listed with a potentially positive registration status that will allow for subsequent analyses of the trial results.

We can only assume that the total numbers of publications identified in MEDLINE and ClinicalTrial.gov are representative of overall research activity in uro-oncology because searching these databases is a very complex process, and many studies are not published due to a number of reasons [6,9,10]. There are certainly additional studies that have not been published in MEDLINE–indexed journals. Searching additional databases, such as Embase or the Cochrane Central Register of Controlled Trials, might be necessary to identify additional related studies. However, some studies might only be identifiable by hand-searching journals that do not publish in English [11-13]. Additionally, we want to point out that the crude number of trials is certainly not associated with the overall quality of the reported trials published in MEDLINE. A possible limitation of our evaluation of the registered studies in ClinicalTrials.gov is the fact that the investigators must register their trials and must update the trial registrations regularly. Thus, we cannot guarantee that our results are actually relevant. However, the International Committee of Medical Journal Editors began promoting trial registration in 2005 and defined July 1, 2005 as the key date for prospective trial registration (http://www.icmje.org). A recent evaluation found that the vast majority of published trials in the field of urology (83%) have been registered since 2006 [14]. Therefore, we are confident that our evaluation considers a representative overview of the international landscape of uro-oncological study.

Whether scientific research on urinary bladder cancer is not as currently interesting as research on renal or prostate cancer remains to be determined. From an economic point of view, research on bladder neoplasms should be extremely important because the therapy and follow-up in this patient cohort are extremely expensive [3]. Approximately 75–85% of patients with bladder cancer present with superficially disease that does not invade the muscles [15] and is treated by local therapy, such as transurethral resections. Transurethral resection is regarded as the gold standard of treatment [15], and its use has changed very little over the last decades. After decades of research, no biomarkers for recurrence and progression are commercially available with the required sensitivity and specificity [16,17]. Moreover, the typical method for the prevention of recurrence of non-muscle invasive bladder cancer has not changed. Only approximately 20% of bladder cancer patients present with muscle invasive neoplasms that require additional neo-adjuvant or adjuvant chemotherapy [18]. The only true innovation in this field is a second-line cisplatin-containing combination chemotherapy [19]. There have also been no fundamental changes in the performance of artificial urinary diversion in the last decade. Regarding the development of new surgical techniques for advanced bladder cancer, this tumor is clearly overshadowed by the implementation of new surgical treatments for kidney and prostate cancer (i.e., open versus laparoscopic versus robotic approaches) [20,21]. Overall, the pharmaceutical and equipment manufacture markets revolve around prostate and kidney neoplasms almost exclusively (i.e., for medical tumor therapy, androgen suppression therapy, and surgical techniques).

A provoking question arises: Does industry determine uro-oncological research activity, or do uro-oncologists simply have no new, improved scientific approaches to implement? We believe that even when the quality or outcome of diagnostics and therapeutic procedures is good, further improvements and research are still warranted.

## Conclusions

The total number of publications in urologic oncology has been rising continuously over recent years, particularly in the form of randomized trials on prostate and kidney cancers. Strikingly, although bladder cancer is one of the most common malignancies in both men and women, relatively few studies have been conducted on urothelial carcinomas of the bladder. Clinical research on neoplasms of the urothelial bladder must be explicitly addressed and supported.

## Competing interests

The authors declare that they have no competing interests.

## Authors’ contributions

FK, JJM, BK and PJG designed the study. FK performed the systematic literature search. FK and BK performed the statistical analysis. FK, BK; MB, JJM, BW, FSK and DGE analyzed and interpreted the data. FK, BK, FSK and MB drafted the manuscript. BW, JJM, PJG and DGE revised the manuscript for scientific and factual content. All authors read and approved the final manuscript.

## Pre-publication history

The pre-publication history for this paper can be accessed here:

http://www.biomedcentral.com/1471-2490/13/56/prepub
